# The relationships between microbiota and the amino acids and organic acids in commercial vegetable pickle fermented in rice-bran beds

**DOI:** 10.1038/s41598-021-81105-x

**Published:** 2021-01-19

**Authors:** Kazunori Sawada, Hitoshi Koyano, Nozomi Yamamoto, Takuji Yamada

**Affiliations:** 1 Corporate Strategy Office, Gurunavi, Inc., Toho Hibiya Building, 1-2-2 Yurakucho, Chiyoda-ku, Tokyo, 100-0006 Japan; 2 School of Life Science and Technology, Tokyo Institute of Technology, 2-12-1 Ookayama, Meguro-ku, Tokyo, 152-8550 Japan

**Keywords:** Microbial communities, Metagenomics, Metagenomics, Food microbiology, Food microbiology

## Abstract

The microbial community during fermented vegetable production has a large impact on the quality of the final products. Lactic acid bacteria have been well-studied in such processes, but knowledge about the roles of non-lactic acid bacteria is limited. This study aimed to provide useful knowledge about the relationships between the microbiota, including non-lactic acid bacteria, and metabolites in commercial pickle production by investigating Japanese pickles fermented in rice-bran. The samples were provided by six manufacturers, divided into two groups depending on the production conditions. The microbiological content of these samples was investigated by high-throughput sequencing, and metabolites were assessed by liquid chromatography-mass spectrometry and enzymatic assay. The data suggest that *Halomonas*, halophilic Gram-negative bacteria, can increase glutamic acid content during the pickling process under selective conditions for bacterial growth. In contrast, in less selective conditions, the microbiota consumed glutamic acid. Our results indicate that the glutamic acid content in fermented pickle is influenced by the microbiota, rather than by externally added glutamic acid. Our data suggest that both lactic acid bacteria and non-lactic acid bacteria are positive key factors in the mechanism of commercial vegetable fermentation and affect the quality of pickles.

## Introduction

Fermented vegetable pickles, historically produced for food preservation, are now produced on a commercial scale. Studies have targeted pickles such as commercially fermented cucumber pickles^1,2^, cabbage pickles^3,4^, fermented kimchi^5,6^, and fermented region-specific vegetables^7–11^ to clarify the changes in the composition of the microbiota during the production process. Most studies emphasised the abundance and variety of lactic acid bacteria (LAB). The initial microbiota of vegetables consist of various bacteria with only a limited number of LAB; the LAB increase in abundance during fermentation in an anaerobic atmosphere, producing lactic acid and thus lowering the pH^2,12^. Undesirable bacteria, such as Gram-negative bacteria, are generally vulnerable to low pH, which enables LAB to thrive in the pickling environment. Investigation of the effect of non-LAB on vegetable fermentation has mostly concerned spoilage^13,14^, and few studies describe positive effects of such bacteria on the quality of the products^15^.

A Japanese fermented pickle called *nukazuke* is made by pickling vegetables with rice-bran mixed with salt and other optional ingredients such as spices, fruit peels, or sodium glutamate. The fermented rice-bran bed contains a well-balanced mixture of Gram-positive and Gram-negative bacteria as well as yeast, which generates favourable flavour in the product pickles^16^. Previously, by culture-dependent methods, LAB were shown to be the main bacteria when using a long-aged rice-bran bed as a seed culture^17^, and on fermenting raw rice-bran bed spontaneously in laboratory conditions^18^. However, the roles of non-LAB, including Gram-negative bacteria, in rice-bran beds have not been fully evaluated. Furthermore, the transitions of the microbiota and metabolites in rice-bran beds during commercial fermentation are still unclear. The fermentation profiles during the pickling process are suggested to be different between homemade samples and industrial samples^6^, which highlights the importance of analysing samples obtained from industrial manufacturers to find knowledge to improve commercial production.

This study aimed to clarify the relationships between microbiota and amino acids and organic acids in rice-bran beds. The pickles were produced by industrial manufacturers under two different conditions, which are expected to influence bacterial growth: condition C pickles were produced in a colder district; and condition W pickles were produced in a warmer district. The difference in production conditions is assumed to result in different microbiota, which is expected to illuminate the relationship between microbiota and metabolites. The results from this study suggest that halophilic Gram-negative bacteria influence glutamic acid production during the pickling process, which indicates the possibility of an important role of non-LAB in improving pickling quality.

## Results

### Salinity during the pickling process

The salinity of the final samples was measured to identify differences in pickling conditions. The mean salinity and standard error were 3.6% ± 0.2% in condition C and 10% ± 1.7% in condition W, a statistically significant difference (*p* = 0.004; Supplementary Information Table S1).

### The difference in the pickling process and the effect on the microbiota

**Figure 1:**
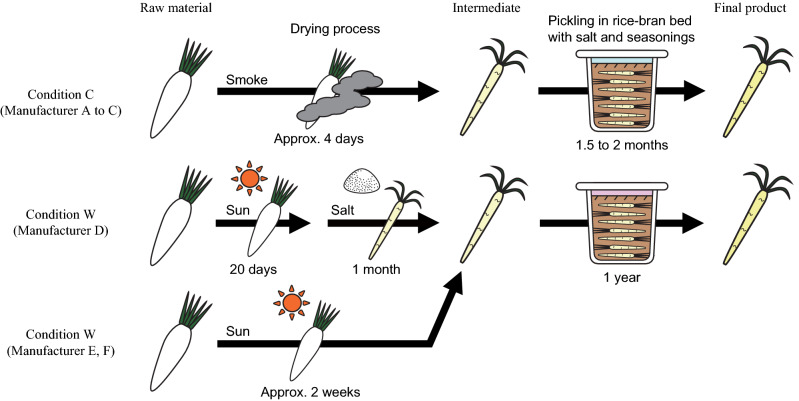
Model of pickling processes using rice-bran bed. Arrows indicate processes. The duration of the process is shown below the arrow. The time stated is based on information provided by the pickle manufacturer.

In both conditions (C and W), dehydrated vegetables were pickled in a rice-bran bed, but the drying method of the vegetables and the temperature during the pickling process were different (Fig. 1). In condition C, the raw vegetables were dehydrated by smoking and then the resulting intermediates were pickled in rice-bran beds during winter (between − 2 and 1 °C on average). In condition W, the raw vegetables were dehydrated under the sun and then these intermediates were pickled through the year (between 6 and 27 °C on average).

**Figure 2:**
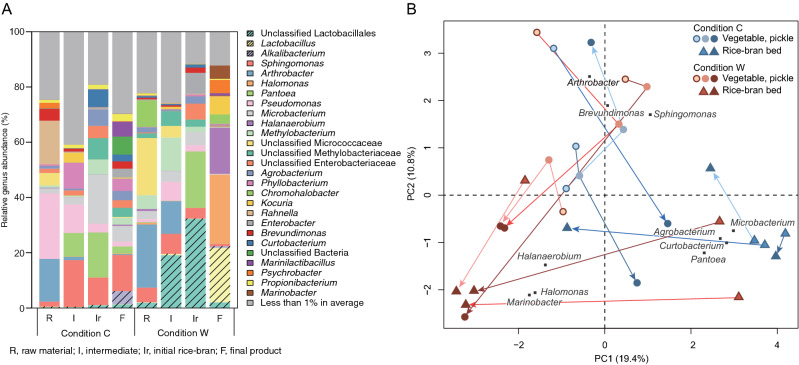
Overview of microbiota in samples. (**A**) Average genus composition at each sampling point in the fermentation process. Genera with a relative abundance of < 1% have been combined. Shaded areas indicate the relative abundance of lactic acid bacteria. (**B**) Principal component analysis of microbiota in 29 samples; circles, vegetables and pickles; triangles, rice-bran bed samples; blue, condition C; red, condition W. Plots for raw materials and the initial rice-bran beds are emphasised by borders. The arrows describe the direction of change in microbiota through the production process. Plots from the same manufacturer are bound by the same line colour. The top 10 genera (marked with squares) were chosen by the length of the loading vector.

To investigate the effect of the difference in conditions, the changes of microbiota were analysed using 16S rRNA gene sequences. In total, 3,042,136 reads were obtained from 29 samples (see Table 1 for details of the sample sizes) after mitochondria- and chloroplast-associated reads were removed. The reads contained 4,414 amplicon sequence variants (ASVs), representing 714 genera based on Greengenes database annotation. The average relative abundance of microbiota at the genus level in equivalent samples is shown in Fig. 2A, and details for each sample are shown in Supplementary Information Fig. S1. Bray–Curtis distances of microbiota were calculated for beta-diversity analysis (Supplementary Information Fig. S2). Permutational multivariate analysis of variance (PERMANOVA) tests showed that the differences between the two conditions in terms of the microbiota in the raw materials (*p* = 0.221), intermediates (*p* = 0.197), and initial rice-bran (*p* = 0.129) were not significant, but the difference in the final products was significant (*p* = 0.005). Thus, the pickling conditions resulted in significant differences in the microbiota, but the district from which the vegetables were harvested and the method by which they were dehydrated did not result in significant differences in the microbiota at those stages of the process. In condition C, the microbiota in the final products belonged to a wide variety of genera, but there was less variety in condition W. Among the predominant genera in condition W were *Lactobacillus*, *Halomonas* and *Halanaerobium*. Halophilic genera made up > 50% of the final microbiota, consistent with the higher salinity in condition W (Fig. 2A; Table S1). Principal component analysis (PCA) described in more detail the changes in the microbiota during the production process (Fig. 2B). The final samples from condition C were plotted in all quadrants. In contrast, the final samples from condition W were plotted in the third quadrant; *Halomonas*, *Marinobacter* and *Halanaerobium* were key components distinguishing the microbiota at this stage from the other sampling points.

**Table 1:** Number of samples obtained from commercial pickle manufacturers.

Condition	Raw material	Intermediate	Initial rice-bran bed	Final product	
				Pickle	Rice-bran bed
Condition C	3	2^a^	3	3	3
Condition W	3	3	3	3	3

^a^An intermediate sample was not provided by Manufacturer B.

### Changes of concentrations of free amino acids and organic acids during the pickling process

**Figure 3:**
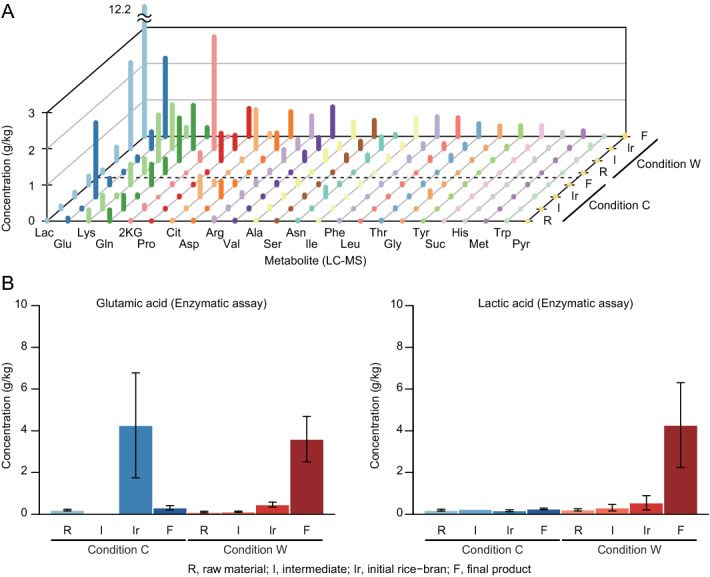
Concentrations of free amino acids and organic acids. (**A**) The concentrations of free amino acids and organic acids analysed by liquid chromatography-mass spectrometry. R, raw material; I, intermediate; Ir, initial rice-bran; F, final product. (**B**) Average glutamic acid and lactic acid concentrations in samples determined by enzymatic assay. Blue bars, condition C; red bars, condition W. Sample abbreviations are the same as in panel (**A**). Error bars indicate standard error (except for the condition C intermediate sample, for which *n* = 2).

Liquid chromatography-mass spectrometry (LC–MS) was used to observe changes in the concentrations of 24 free amino acids and organic acids during the picking process. An overview of the results is shown in Fig. 3A, and the raw data are shown in Table S2. Comparison of the concentrations of free amino acids and organic acids in the final products was performed with the average value from final pickles and final rice-bran beds, because the content of the final pickle and rice-bran bed is reasonably assumed to be in equilibrium. The results show a prominent difference in the concentrations of glutamic acid and lactic acid between conditions C and W. Glutamic acid and lactic acid were the top two metabolites (in terms of final concentration) in condition W but not in condition C. The glutamic acid and lactic acid concentrations markedly increased during the production process in condition W. Contrast to condition W, the glutamic acid concentration decreased from that in the initial rice-bran bed to the final product in condition C. The concentration of the other free amino acids and organic acids was almost unchanged, which suggested that glutamic acid was not diluted by the water from the vegetables but was transformed into other compounds chemically or biologically. To determine the changes in glutamic acid and lactic acid concentrations more precisely, enzymatic analysis which enables evaluation of samples directly after extraction was performed. The results from enzymatic analysis (average, Fig. 3B; raw data, Table S3) were consistent with the results from LC–MS. Glutamic acid was degraded almost completely from the initial rice-bran bed to the final product in condition C, while the glutamic acid and lactic acid concentrations were increased in the final product in condition W. In condition W, from the intermediate state, the glutamic acid concentration was increased by 31-fold and the lactic acid concentration was increased by 13-fold in the final products; from the initial rice-bran bed, both the glutamic acid and lactic acid concentrations were increased by 7.8-fold in the final products. Significance of the difference in the glutamic acid and lactic acid concentrations in the final products between conditions C and W was tested using the Wilcoxon rank sum test, which indicated that the glutamic acid and lactic acid concentrations were significantly different (*p* = 0.02 for glutamic acid, *p* = 0.01 for lactic acid).

### Correlation between bacterial relative abundance and glutamic acid and lactic acid concentrations

**Figure 4:**
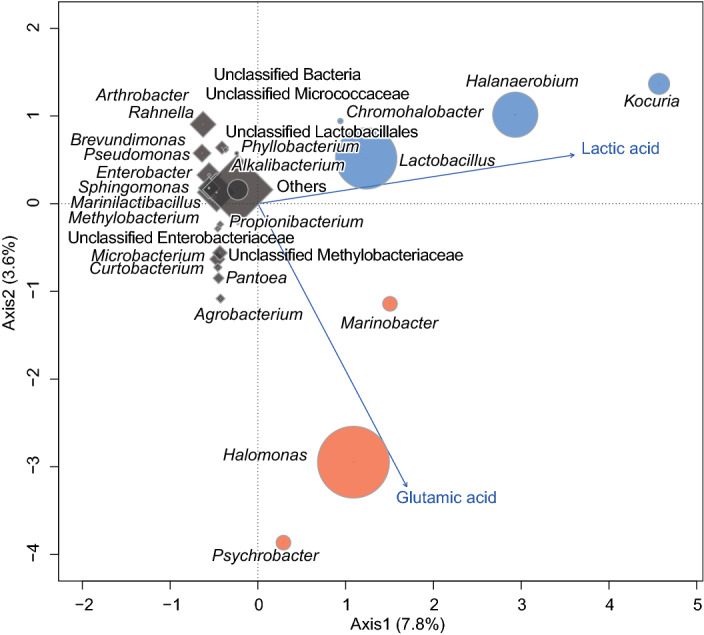
Canonical correspondence analysis between genera showing > 1% relative abundance in total, and glutamic acid and lactic acid concentrations. Arrows represent metabolites. Diamonds and circles represent genera found in conditions C and W, respectively. The size of a marker represents the relative importance of the genus calculated as log of the ratio of relative abundance in the raw material to that in the final product multiplied by the relative abundance in the final product. Red markers represent genera potentially correlated with glutamic acid concentration. Blue markers represent genera potentially correlated with lactic acid concentration. Grey markers represent genera irrelevant to glutamic acid and lactic acid concentrations. Values in parentheses beside the axis label indicate the variability of the axis.

**Figure 5:**
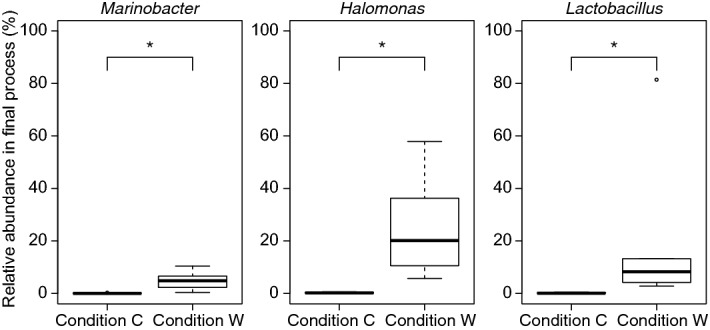
Distribution of relative genus abundance in the final product. The title of each boxplot shows the genus name as annotated using the Greengenes database. Asterisk(*) indicates a significant difference (*p* < 0.05) detected by ANalysis of COmposition of Microbiomes (detection ratio: 0.8).

Canonical correspondence analysis (CCA) can be used to identify correlations between microbiota and metabolites^19^. The results of such analysis for our data indicated that *Halomonas*, *Marinobacter* and *Psychrobacter* (shown as red bubbles in Fig. 4) were potentially correlated with glutamic acid concentration. *Kocuria*, *Halanaerobium*, *Lactobacillus* and *Chromohalobacter* (shown as blue bubbles in Fig. 4) were potentially correlated with lactic acid concentration. The low variability of constrained axes, i.e. 7.8% for Axis 1 and 3.6% for Axis 2, can be explained by a limited number of genera correlating with glutamic acid and lactic acid concentrations. For further identification of genera that influenced glutamic acid and lactic acid concentrations, correlation analysis using relative genus abundance and enzymatic assay was performed. The results (Table 2) showed that the relative abundance of *Marinobacter* and *Halomonas* correlated positively with the glutamic acid concentration in condition W. The relative abundance of *Kocuria* and *Halanaerobium*, which are non-LAB, positively correlated with the lactic acid concentration in condition W. These genera may play a key role in the increasing glutamic acid and lactic acid concentrations in condition W. No genus showed a negative correlation between its relative abundance and the glutamic acid concentration in condition C. For confirmation of these findings, ANalysis of COmposition of Microbiomes (ANCOM) was performed to reveal significantly abundant genera in the final products. Three genera such as *Marinobacter*, *Halomonas* and *Lactobacillus* were significantly abundant in the final product of condition W (Fig. 5). The results discussed above suggested that *Marinobacter* and *Halomonas* may relate to glutamic acid content during the pickling process. No genus showed significant abundance in condition C. Thus, neither correlation analysis nor ANCOM could show the results corresponding to CCA from condition C.

**Table 2:** Significant correlations between the relative abundance of genera and glutamic acid and lactic acid concentrations during the pickle production process.

Condition	Genus	Metabolite	Correlation coefficient
Condition C	No genus detected		
Condition W	*Marinobacter*	Glutamic acid	0.51
	*Halomonas*	Glutamic acid	0.87
	*Psychrobacter*	Glutamic acid	0.81
	*Kocuria*	Lactic acid	0.72
	*Halanaerobium*	Lactic acid	0.60

Based on the results described above, there was a possibility that the decrease of glutamic acid observed during the process in condition C was caused by nonbiological factors. PICRUSt analysis was performed using relative genus abundance in the final products to evaluate the metabolic features of the microbiota to help deduce the reason for the decreasing glutamic acid concentration in condition C. PICRUSt analysis (Table 3) identified 13 Kyoto Encyclopedia of Genes and Genomes (KEGG) ortholog groups^20^ showing significant differences between conditions C and W in the gene content of the microbiota in the final product. Among them, the relative abundance of all ortholog groups annotated to glutamic acid transporters was higher in condition C than in condition W. This result suggested that the microbiota in condition C had relatively higher ability for glutamic acid transport than those in condition W, which enhanced the intake and consumption of glutamic acid in condition C.

**Table 3:** Kyoto Encyclopedia of Genes and Genomes ortholog groups identified by PICRUSt analysis as relevant to glutamic acid showing significant differences in relative abundance between conditions C and W.

Ortholog	Average of relative abundance		Ratio^a^ (W/C)	Description
	Condition C (%)	Condition W (%)		
K14940	0.0005	0.0015	3.126	Alpha-l-glutamate ligase
K05844	0.0107	0.0322	2.995	Ribosomal protein S6–l-glutamate ligase
K00262	0.0193	0.0408	2.113	Glutamate dehydrogenase (NADP+)
K00261	0.0089	0.0029	0.320	Glutamate dehydrogenase (NAD(P)+)
K01458	0.0053	0.0008	0.156	*N*-formylglutamate deformylase
K10004	0.0114	0.0016	0.140	Glutamate/aspartate transport system ATP-binding protein
K10003	0.0106	0.0015	0.138	Glutamate/aspartate transport system permease protein
K10002	0.0106	0.0015	0.138	Glutamate/aspartate transport system permease protein
K10001	0.0224	0.0029	0.127	Glutamate/aspartate transport system substrate-binding protein
K01295	0.0245	0.0021	0.085	Glutamate carboxypeptidase
K18653	0.0007	0.0000	0.067	3-dehydro-glucose-6-phosphate-glutamate transaminase
K12941	0.0137	0.0007	0.049	Aminobenzoyl-glutamate utilisation protein B
K01461	0.0006	0.0000	0.045	*N*-acyl-d-glutamate deacylase

Statistical significance was determined using the Wilcoxon rank sum test (*p* < 0.05).

^a^Values calculated by dividing the average of relative abundance in condition W by that in condition C.

## Discussion

This study examined the effects of microbiota derived from the production of commercial pickles on the content of free amino acids and organic acids in the pickles. The pickles manufactured in Akita (condition C) were made at colder temperature with smoke-dried vegetables, while pickles made in Aichi (condition W) were produced at warmer temperature using sun-dried vegetables.

Highly diverse microbiota were found in the final product from condition C compared with condition W. This result is presumably due to differences between conditions C and W. On one hand, the salinity and lactic acid content in the final products in condition C were significantly lower than those in condition W, which would enable most species of bacteria to grow without inhibition. On the other hand, features of condition C include low temperature and chemical substances generated by smoking, such as phenols and ketones^21^, which generally repress the growth of bacteria. Based on the fact that the most abundant genus in the final products from condition C was *Sphingomonas*, an aerobic genus, the repression of bacterial growth would contribute to keep the conditions aerobic. The maintenance of relatively aerobic conditions may favour greater bacterial diversity. The high content of glutamic acid in the initial rice bran of condition C was also a difference from condition W. This difference could help various bacteria, especially glutamate auxotrophic bacteria, to survive during the final process under condition C. Glutamic acid is an important amino acid used as a substrate for protein synthesis and transamination^22^. The results from PICRUSt analysis showing a higher abundance of transporter genes for glutamic acid in the microbiota of the final products in condition C may indicate the survival of glutamate-requiring bacteria. It thus appears that a combination of conditions, i.e., a high concentration of glutamic acid, low salinity, moderate pH, and an aerobic environment, leads to a higher diversity of microbiota in the final products from condition C.

However, condition W, with a higher level of salinity, selects for halotolerant bacteria, which decrease the oxygen level, promoting lactic acid fermentation as a result of anaerobic respiration. The lactic acid produced decreases the pH, which selects further for acid-tolerant bacteria, making the microbiota less diverse. These findings correspond to those in a previous study in which a wide variety of bacterial families in the initial rice-bran bed gradually decreased and only a limited number of families survived in warm conditions after fermentation for 2 months^18^.

The microbiota in the final product in condition W included several predominant genera, especially halophilic bacteria, that would affect the glutamic acid concentration, which was found to increase during the production process; their relative abundance was due to the environmental conditions including warmer temperature. CCA, correlation analysis and ANCOM suggested that *Halomonas* plays an important role in the increase of glutamic acid concentration in the pickles in condition W. Previous study implicated Gram-negative bacteria in adding complex flavours during the pickling process using a rice-bran bed^16^. The present study suggests for the first time that *Halomonas* is relevant to the increase of glutamic acid, which is an umami substance in fermented pickles. The differences in final glutamic acid concentration in conditions C and W indicate that the concentration of glutamic acid in fermented pickles is influenced by the microbial community, rather than by directly added glutamic acid. This suggestion further emphasises the importance of the microbiota for the quality of fermented pickles. *Halomonas* has been studied mainly in terms of bioremediation and metabolic engineering^23^ and there are limited studies about this genus in fermented foods or amino acid production. *Halomonas* was reported to be dominant in Korean fermented shrimp^24^ and Chinese dry fish^25^. In another study, *Halomonas* were found on the surface of cheese, but their effect on the quality of cheese is still unclear^26,27^. Glutamic acid is produced from 2-oxoglutaric acid in the tricarboxylic acid cycle by glutamate dehydrogenase (GDH) using either NADH or NADPH as a coenzyme. All *Halomonas* bacteria available in the KEGG pathway database have GDH that can use NADH as a coenzyme^28^. This feature is advantageous in condition W, because glutamic acid production accompanies the consumption of excess NADH in anaerobic conditions. The present study suggests the novel possibility of halophilic bacteria improving the quality of vegetable fermentation by affecting the glutamic acid content during pickling.

The relationship between lactic acid production and LAB was not clear in this study. The pickles in condition C contained only a small amount of lactic acid, which made the analysis very difficult. Only CCA suggested the involvement of *Lactobacillus* in lactic acid production, and no other analysis indicated correlation between LAB and lactic acid concentration. Moreover, analyses of the microbiota in condition W showed the possibility that *Halanaerobium* and *Kocuria*, non-LAB, produce lactic acid. This result does not correspond with studies in which *Lactobacillus* were predominant in rice-bran beds and lactic acid fermentation was performed by LAB, especially *Lactobacillus*^17,18,29^. *Kocuria* is the less likely to produce lactic acid during the pickling process because it is an aerobic genus^30^ but *Halanaerobium*, an obligately anaerobic genus, may perform anaerobic respiration to produce lactic acid^31^ during pickle production. Our results may be a novel finding about *Halanaerobium*, but there is still the possibility of an artefact. Previous study indicated that the microbiota of pickles fermented using rice-bran beds change, decreasing the relative abundance of LAB toward the end of the production process^16^. Our samples of final products in condition W may have been taken from the tanks after complete lactic acid fermentation.

The limitations of interpreting the results of correlation analysis should be mentioned. The relative abundance of genera does not describe the actual microbial population but the proportion of the total population of living cells and dead cells occupied by that genus. In addition, the samples used in this study were taken only at the beginning and the end of pickling, but not in between. Therefore, the suggestion that a specific genus produces a specific metabolite based on the results of correlation analysis is hypothesis. However, only the final step of the pickle production process involved fermentation, and samples from the other steps (e.g., raw vegetables and intermediate [dried] vegetables) may reasonably be expected to contain smaller populations of bacteria than the samples from final step. Also, the physiological features of the genera described above support the suggested correlations between genera and metabolites. Thus, the interpretations given in this study are expected to be meaningful to some extent. Further analysis, such as culture-dependent study with isolated bacteria from pickles, is needed to evaluate the interpretations given above.

This study indicates the possibility that halophilic bacteria influence glutamic acid content by comparing fermented pickles produced in rice-bran beds in different conditions. The science of vegetable fermentation still requires a lot more investigation because the microbiota vary greatly depending on the conditions used for production, the vegetables used, and the salinity. The findings in this study suggest the idea that not only LAB but also other bacteria must be investigated to determine the whole mechanism of the pickling process.

## Materials and methods

### Sample collection and processing

Samples were provided by manufacturers from (i) Akita Prefecture, a northern, colder district in Japan (Manufacturers A to C); and (ii) Aichi Prefecture, a central, relatively warmer district in Japan (Manufacturers D to F). The samples included Japanese white radish (raw material), dehydrated radish (intermediate), the initial rice-bran bed, and the final pickle and rice-bran bed, produced in winter 2017 to 2018 (Table 1). In this study, the final product is defined as the final pickle and the final rice-bran bed. The chosen manufacturers make their pickles using locally grown radish. Pickles were produced as shown in Fig. 1. Briefly, in the Akita procedure (condition C), the raw materials were first dried by smoke without using salt. The intermediates were then pickled in a seasoned rice-bran bed without temperature control. In the Aichi procedure (condition W), the raw materials were first sun-dried, then pickled in a seasoned rice-bran bed without temperature control. Manufacturer D (who used condition W) performed a salting process before pickling in the rice-bran bed, but the other manufacturers used no salt for the drying process. The duration of the pickling process was that used in the conventional production procedure of each respective manufacturer.

Bacterial cells were collected from all samples for DNA extraction. The peeled skin of raw materials, intermediates, and final pickles was cut into approximately 140 cm^2^ pieces in aseptic conditions, then washed vigorously in 10 mL sterile 0.85% NaCl solution for 15 s. Debris was subsequently removed from the washing liquid by passing it through a 40-µm pore-size filter. Next, the filtered solution was centrifuged at 8000 × *g* at 4 °C for 10 min, after which the bacterial cells were collected. Cells were also collected from approximately 3 g of the initial and final rice-bran bed by applying the same method. Cells were stored at − 20 °C until DNA extraction was performed. The salinity of the final pickle and rice-bran bed was measured with squeezed juice of the pickle and washing solution of the rice-bran bed prepared by adding ultrapure water, respectively. The juice and the washing solution were applied to an LAQUAtwin-Salt-22 ion meter (Horiba, Kyoto, Japan) to determine the salinity at least in duplicate. The statistical significance of the difference in salinity between two conditions was analysed using the Wilcoxon rank sum test (*p* < 0.05).

## DNA extraction and 16S rRNA gene sequencing

The collected cells were resuspended with 1 mg mL^−1^ lysozyme (Wako Pure Chemicals, Tokyo, Japan) in Tris–EDTA buffer and incubated at 37 °C for 5 min. DNA was then extracted from the processed cells using a Genomic DNA Clean and Concentrator Kit (Zymo Research, Irvine, CA, USA). The V3–V4 region of the 16S rRNA gene was PCR-amplified from the extracted DNA using universal primers with Illumina MiSeq (Illumina, San Diego, CA, USA) barcode and adaptor sequences^32^. The PCR conditions were: initialisation at 94 °C for 30 s; followed by 30 cycles of denaturation at 94 °C for 15 s, annealing at 50 °C for 30 s, and extension at 72 °C for 1 min; then final extension at 72 °C for 5 min. The size and concentration of the amplified DNAs was analysed using an Agilent DNA High Sensitivity DNA Kit and an Agilent 2100 Bioanalyzer (Agilent Technologies, Santa Clara, CA, USA), after which equimolar amounts of DNA from each sample were loaded into the Illumina MiSeq system. The raw FASTQ files for the 29 samples (Table 1) were deposited in the DNA Database of Japan (DDBJ) under DRA accession number DRA009842, and were associated with the BioProject titled “Microbiota in fermented pickles with rice-bran bed” under BioProject accession number PRJDB9501.

### Processing 16S rRNA sequence data for analysis of microbiota

First, reads containing N(s) were removed, after which PhiX reads identified by bowtie2^33^ (version 2.1.0) were eliminated, and finally unpaired reads were deleted using in-house scripts. Processed reads were then filtered, denoised and merged using the dada2^34^ functions in the R program (version 1.8). In dada2, the forward and reverse reads were truncated to 270 bp and 220 bp respectively, after which the primer sequences were trimmed. Reads with a labelled Phred score < 5 at the first nucleotide or an expected error^35^ > 2 for a forward read or > 5 for a reverse read were removed. After the remaining reads were denoised and merged, an ASV^36^ table was constructed, and chimeric reads were removed. The resulting ASV table and reads were imported into QIIME 2^37^ (version 2018.11) for further analysis. Default values were used for all optional parameters in the QIIME 2 commands. Taxa were assigned to ASVs at the genus level using the Greengenes (version 13.8) database^38^. ASV sequences for mitochondria and chloroplasts were removed from the sequence table. An overview of microbiota in the condition was described by calculating the average relative abundance at each sampling point. Similarity of microbiota at the genus level was illustrated by PCA using R. Beta diversity analysis was performed using a built-in function in QIIME 2. Reads from all samples were rarefied to 7997, which was the smallest read in the dataset, then we calculated the Bray–Curtis distance of microbiota at the genus level. Significant difference in microbiota between two conditions was analysed by PERMANOVA test (*p* < 0.05). ANCOM^39^ was used to detect significant differences in the relative abundance of genera between conditions using the Wilcoxon rank sum test with taxa-wise multiple testing correction (*p* < 0.05). The detection ratio was set to 0.8. Finally, PICRUSt analysis was performed to evaluate the potential ability for glutamic acid intake and metabolism in microbiota^40^ using a QIIME 2 (version 2019.7) plug-in module with default values for optional parameters except the hidden-state prediction method, which was set to ‘mp’. The output file of KEGG ortholog metagenome predictions was used for further analysis. The relative abundance of KEGG orthologs which are relevant to glutamic acid was calculated from the gene content to detect significant difference between two conditions using the Wilcoxon rank sum test (*p* < 0.05).

### Measurement of concentrations of free amino acids and organic acids by LC–MS and enzymatic assay

Raw materials, intermediates and final pickles were roughly chopped, after which approximately 1-g aliquots were homogenised in an equivalent amount (w/w) of ultrapure water using a high-power homogeniser (ASONE, Tokyo, Japan) at 3000 rpm for 5 min on ice. The homogenate was then centrifuged at 20,400×*g* at 4 °C for 5 min, after which the supernatant was reserved for analysis. Rice-bran bed samples were also homogenised using the same method, but the level of dilution (w/w) was changed between 1 and 5 depending on the water content of the rice-bran bed sample. Then, 40 µL of the supernatant were mixed with 10 µL of internal standard solution (5 g L^−1^ methionine sulfone). Next, 175 µL of deproteinisation solution consisting of methanol and chloroform (2.5:1, v/v) were added and mixed vigorously. The mixture was subsequently incubated with vigorous shaking at 37 °C for 30 min, then centrifuged at 20,400×*g* at 4 °C for 5 min. Next, 200 µL supernatant were mixed well with 88 µL ultrapure water and centrifuged at 20,400×*g* at 4 °C for 5 min. Finally, 200 µL supernatant were mixed well with 100 µL ultrapure water and passed through a 0.45-µm pore-size filter to remove insoluble particles. The final aqueous solutions were analysed using an LCMS-8050 system (Shimadzu, Kyoto, Japan) with a Discovery HS F5-3 column (Sigma-Aldrich, St Louis, MO, USA). The analysis was performed using the Primary Metabolite Ver. 2 method package (Shimadzu). The conditions for liquid chromatography were: mobile phases, solution A, 0.1% formic acid in water Optima LC/MS (Fisher Chemical, Waltham, MA, USA), and solution B, 0.1% formic acid in acetonitrile Optima LC/MS (Fisher Chemical); flow rate, 0.25 mL min^−1^ in gradient mode; gradient program, 100% solution A at 0–2 min, 100%–75% solution A at 2–5 min, 75%–65% solution A at 5–11 min, 65%–5% solution A at 11–15 min, and 5% solution A at 15–20 min; column oven temperature, 40 °C. The conditions for mass spectrometry were: ionisation mode, electrospray ionisation; analysis mode, multiple reaction monitoring; nebuliser gas flow rate, 3.0 L min^−1^; drying gas flow rate, 10 L min^−1^; heating gas flow rate, 10 L min^−1^; interface temperature, 300 °C; desolvation line temperature, 250 °C; and heat block temperature, 400 °C. The external standard was used to calculate the concentration of the compound in mg L^−1^. The internal standard was used to estimate the dilution rate and experimental error. The concentration of each compound was transformed into mg kg^−1^ based on the sample weight. The density of homogenised solid samples was assumed to be 1 g mL^−1^.

Enzymatic assay was performed to determine the concentration of glutamic acid and lactic acid using the supernatant of homogenised sample before deproteinisation in duplicate. Glutamic acid concentration was assayed using a YAMASA NEO l-glutamate Assay Kit (Yamasa, Chiba, Japan) and lactic acid concentration was measured using an F-kit Lactic acid (Roche, Basel, Switzerland) following the instructions from the manufacturers. An overview of transition of the concentrations of free amino acids and organic acids was obtained by calculating the average at each sampling point. The statistical significance of difference between two conditions in terms of glutamic acid or lactic acid concentration was tested by Wilcoxon rank sum test (*p* < 0.05).

### Correlation between relative genus abundance and metabolite concentration

Further statistical analysis was performed to detect correlations between the relative abundance of a genus and glutamic acid and lactic acid concentrations determined by enzymatic assay, using R. Genera were excluded from the correlation analysis if their relative abundance was < 1% on average during the whole production process because minor genera should have little effect on metabolite concentrations. CCA was performed using vegan library^41^. Correlation coefficients and correlation significance were calculated by the Pearson method. Significant correlations (*p* < 0.05) were examined to select meaningful correlations.

## Supplementary Information

Supplementary Information.

## Data Availability

The datasets generated during 16S rRNA sequencing are available in the DDBJ/EMBL/GenBank databases under accession number DRA009842. All data generated during LC–MS analysis and enzymatic assay are included in this article.
