# Supplementary material for: The relationships between microbiota and the amino acids and organic acids in commercial vegetable pickle fermented in rice-bran beds
Source: Sci Rep. 2021 Jan 19;11:1791. doi: 10.1038/s41598-021-81105-x (PMC7815776; doi:10.1038/s41598-021-81105-x)
Supplement: Supplementary file 1 — Supplementary Information. [file 41598_2021_81105_MOESM1_ESM.pdf]

The relationships between microbiota and the amino acids and organic acids in commercial vegetable pickle fermented in rice-bran beds

Kazunori Sawada,[a] Hitoshi Koyano,[b] Nozomi Yamamoto,[b] Takuji Yamada[b,]*

[a] Corporate Strategy Office, Gurunavi, Inc., Toho Hibiya Building, 1-2-2 Yurakucho, Chiyoda-ku, Tokyo, 100-0006, Japan

[b] School of Life Science and Technology, Tokyo Institute of Technology, 2-12-1 Ookayama, Meguro-ku, Tokyo, 152-8550, Japan

Kazunori Sawada sawada-ka@gnavi.co.jp

Hitoshi Koyano koyano1917@gmail.com

Nozomi Yamamoto nozomy77@gmail.com

Takuji Yamada takuji@bio.titech.ac.jp

*Corresponding author takuji@bio.titech.ac.jp

Supplementary Figures

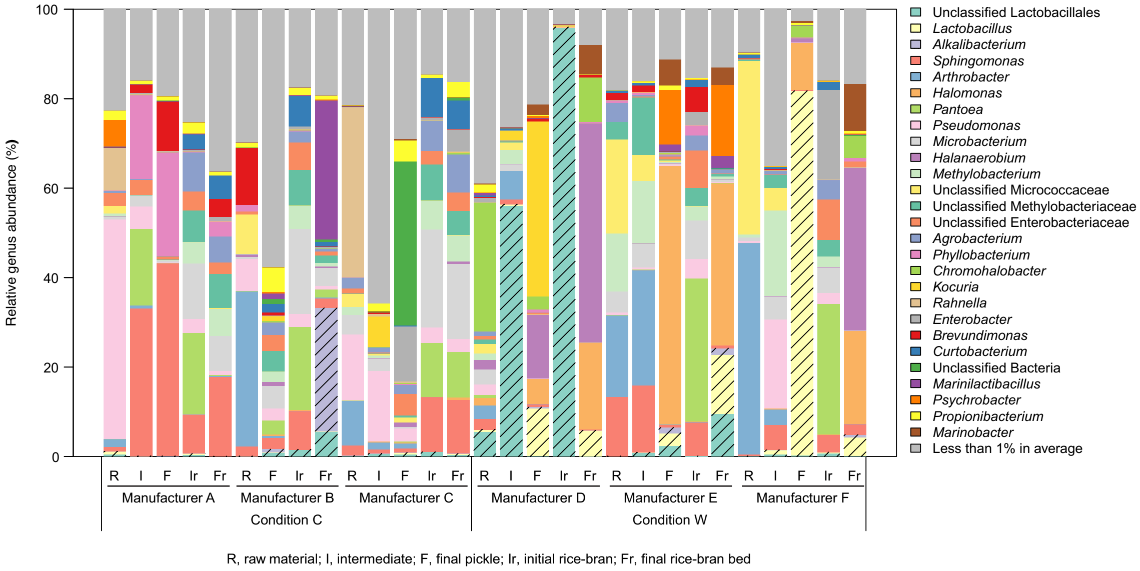

Fig. S1. Genus composition in each sample. Genera with relative abundance <1% have been combined. Shaded areas indicate the relative abundance of lactic acid bacteria.

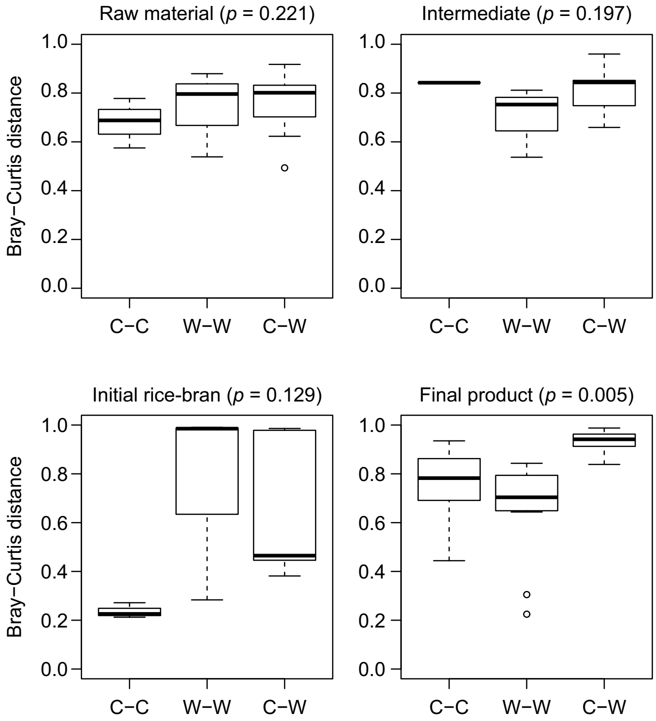

Fig. S2. Bray-Curtis distance of each sample group for beta-diversity analysis. C–C, intragroup distance of condition C; W–W, intragroup distance of condition W; C–W, intergroup distance between conditions C and W. The *p*-values calculated by PERMANOVA test to evaluate the significance of the difference in the microbiota between condition C and W are indicated in parentheses above the plot area.

Supplementary Tables

Table S1. Salinity of final products.

	Condition C			Condition W		
	Manufacturer A	Manufacturer B	Manufacturer C	Manufacturer D	Manufacturer E	Manufacturer F
Salinity of pickle (%)	4.2	3.8	4.1	11.0	7.0	9.4
Salinity of rice-bran bed (%)	2.6	3.4	3.4	9.4	4.2	16.8
Average ± S.E.	3.6 ± 0.2			10 ± 1.7		
p-value	0.004					

S.E., standard error. The Wilcoxon rank sum test was used to calculate the *p*-value.

Table S2. Raw data on free amino acid and organic acid concentrations determined by LC-MS.

(Unit: mg/kg)	Condition C													
	Manufacturer A					Manufacturer B				Manufacturer C				
Metabolite	Raw material	Intermediate	Final pickle	Initial rice-bran bed	Final rice-bran bed	Raw material	Final pickle	Initial rice-bran bed	Final rice-bran bed	Raw material	Intermediate	Final pickle	Initial rice-bran bed	Final rice-bran bed
Alanine	22.0	58.5	171.7	74.3	129.8	18.9	159.4	39.9	203.2	31.1	90.2	119.6	58.7	132.9
Arginine	2.2	29.4	177.5	171.7	121.5	190.4	97.2	85.6	146.2	81.3	95.3	108.0	109.1	128.0
Asparagine	9.2	28.1	11.9	199.0	2.8	40.1	84.3	349.5	96.7	39.5	42.4	75.1	265.1	100.0
Aspartic acid	4.9	21.4	195.8	398.4	148.3	37.9	73.1	501.8	94.7	37.1	41.3	36.9	384.7	65.6
Glutamic acid	65.5	36.4	317.3	4554.7	375.5	76.0	91.1	811.4	127.2	104.9	24.3	67.0	780.5	131.4
Glutamine	123.4	376.3	661.7	86.7	340.9	396.3	644.0	32.4	760.0	409.3	378.0	660.1	60.1	714.6
Glycine	15.0	11.7	102.9	506.3	77.4	4.0	19.0	8.4	26.9	6.0	11.6	10.8	6.8	13.7
Histidine	3.1	9.7	24.6	24.3	23.9	17.1	11.8	18.0	8.8	14.1	11.0	16.5	16.1	18.4
Isoleucine	6.8	28.1	115.5	22.1	78.4	35.7	34.0	11.1	20.6	43.8	39.3	68.1	14.1	85.8
Leucine	1.0	8.8	83.2	60.4	59.9	5.6	14.9	9.7	13.4	4.3	13.9	27.6	12.3	42.1
Lysine	123.8	378.1	662.5	86.7	350.3	398.6	642.0	33.2	756.8	409.7	383.2	651.1	59.3	716.9
Methionine	1.4	4.5	37.8	24.5	27.2	3.4	7.3	5.8	5.6	2.0	4.2	11.1	5.9	19.2
Phenylalanine	3.5	15.9	76.6	30.5	58.5	29.1	34.7	9.9	49.4	32.3	20.2	32.6	11.5	49.9

Proline	3.3	10.2	26.7	37.0	20.4	11.5	38.2	24.1	64.4	8.3	30.9	14.3	25.4	18.2
Serine	6.8	9.7	67.7	69.1	49.7	37.4	29.8	45.9	34.2	16.2	29.1	20.1	50.1	28.0
Threonine	7.7	27.9	84.1	45.9	63.3	37.1	58.0	30.6	63.5	40.0	45.8	44.7	36.0	43.6
Tryptophan	0.7	2.9	21.7	33.4	18.5	10.7	6.8	28.5	12.0	14.6	8.0	8.8	25.7	15.1
Tyrosine	1.1	8.7	62.1	41.3	50.0	6.2	34.5	24.7	59.3	9.7	15.1	22.1	22.1	40.6
Valine	10.0	32.8	119.7	49.9	79.0	41.8	55.7	21.4	65.2	28.3	40.9	66.8	22.6	94.0
2-Ketoglutaric acid	66.5	135.3	0.0	0.0	0.0	0.0	0.0	0.0	323.2	0.0	82.7	0.0	0.0	240.1
Citric acid	20.7	17.0	201.5	886.2	159.4	38.4	81.2	366.7	69.2	26.2	56.3	30.3	467.0	118.1
Lactic acid	0.0	95.4	440.3	55.1	482.3	38.8	0.0	253.7	359.0	37.9	70.8	140.7	90.8	362.0
Pyruvic acid	0.0	0.0	0.0	15.8	0.0	0.0	0.0	0.0	21.9	0.0	0.0	0.0	21.1	0.0
Succinic acid	3.5	0.9	58.2	91.9	26.9	19.6	61.1	4.7	153.5	3.5	36.8	23.1	6.6	35.1

(Unit: mg/kg)	Condition W														
	Manufacturer D					Manufacturer E					Manufacturer F				
Metabolite	Raw material	Intermediate	Final pickle	Initial rice-bran bed	Final rice-bran bed	Raw material	Intermediate	Final pickle	Initial rice-bran bed	Final rice-bran bed	Raw material	Intermediate	Final pickle	Initial rice-bran bed	Final rice-bran bed
Alanine	22.2	259.5	465.6	279.6	372.8	67.0	497.8	629.7	102.0	519.7	21.4	64.1	266.2	56.1	140.0
Arginine	24.1	252.9	1498.5	376.3	1020.1	109.4	412.9	467.6	231.5	138.1	97.3	267.0	6.1	132.2	319.4
Asparagine	16.7	216.6	16.0	397.7	4.8	35.8	177.8	87.9	494.6	54.4	30.3	90.0	88.8	100.4	126.4
Aspartic acid	30.2	149.2	1670.6	408.5	1142.2	33.8	35.2	559.2	658.6	403.8	6.6	44.2	189.1	238.3	168.5
Glutamic acid	23.3	141.8	3341.4	414.6	3087.1	43.0	148.4	3245.8	746.7	2843.1	69.1	39.6	274.8	232.7	199.4
Glutamine	309.1	2617.7	325.2	3519.2	207.3	521.9	36.0	401.1	60.0	325.6	317.0	886.8	147.4	11.4	90.6
Glycine	3.8	68.2	607.2	85.7	449.9	10.4	42.3	314.5	40.9	231.5	10.0	21.6	99.8	14.0	64.7
Histidine	7.7	80.1	197.1	73.6	158.9	13.7	65.0	140.5	44.7	118.9	16.4	43.3	79.6	13.7	52.8
Isoleucine	6.9	222.5	1203.8	410.5	798.4	11.4	109.8	409.1	23.6	296.9	15.9	60.5	220.3	8.6	163.0
Leucine	3.3	163.1	1335.5	386.4	800.8	3.6	84.1	406.6	29.7	295.7	7.1	25.8	174.8	9.4	102.5
Lysine	319.0	2652.7	330.0	3562.1	237.4	535.0	226.2	406.9	64.8	328.9	322.4	912.8	154.3	9.5	102.5
Methionine	1.9	46.1	382.8	99.5	218.8	4.9	21.4	148.8	17.8	106.5	2.1	15.0	55.4	18.1	40.9
Phenylalanine	3.7	204.1	1482.6	351.9	945.0	6.6	73.9	323.0	47.5	254.2	8.8	40.2	192.7	10.2	120.6

Proline	22.1	1504.1	1356.5	1037.1	808.2	57.7	464.5	1176.5	71.7	934.5	16.6	306.4	360.2	6.1	39.0
Serine	23.2	298.0	1017.0	429.8	674.2	43.0	139.3	432.5	141.8	314.6	11.9	52.1	150.2	46.4	96.1
Threonine	15.6	250.4	805.4	299.6	616.6	20.6	129.8	284.0	59.4	212.7	29.4	70.7	91.7	16.3	131.9
Tryptophan	1.8	73.1	17.7	165.0	15.6	2.3	20.7	47.6	110.1	37.1	5.5	24.9	5.8	62.0	3.7
Tyrosine	2.6	74.0	739.3	115.0	446.1	2.7	30.5	239.7	67.1	181.3	5.7	23.7	114.4	28.6	92.1
Valine	22.6	423.0	2050.5	823.3	1317.6	24.9	199.1	605.3	72.1	427.0	26.9	96.8	319.8	30.8	202.4
2-Ketoglutaric acid	0.0	670.3	0.0	2141.2	0.0	0.0	0.0	0.0	7122.0	0.0	0.0	0.0	0.0	0.0	0.0
Citric acid	11.6	122.4	53.4	1315.9	11.0	21.7	6.7	285.0	1377.3	159.6	16.3	40.2	2.9	536.4	29.4
Lactic acid	16.3	379.4	39678.1	6386.9	22181.4	31.5	745.0	1500.2	744.3	1081.4	196.0	0.0	8760.7	0.0	475.1
Pyruvic acid	5.7	15.7	107.7	0.0	40.9	0.0	0.0	67.4	0.0	14.2	0.0	0.0	0.0	0.0	32.1
Succinic acid	6.9	70.7	801.5	161.7	486.6	1.5	3.6	164.6	19.3	155.3	1.5	30.0	184.3	8.5	12.0

Table S3. Raw data on free amino acid and organic acid concentrations determined by enzymatic assay. The values were the average of technical duplicates.

	Condition C														
(Unit: mg/kg)	Manufacturer A					Manufacturer B				Manufacturer C					
Metabolite	Raw material	Intermediate	Final pickle	Initial rice-bran bed	Final rice-bran bed	Raw material	Final pickle	Initial rice-bran bed	Final rice-bran bed	Raw material	Intermediate	Final pickle	Initial rice-bran bed	Final rice-bran bed
Glutamic acid	122.7	15.5	546.4	9293.8	689.7	195.9	105.2	1659.8	74.2	261.9	37.1	79.4	1829.9	150.5
Lactic acid	174.9	278.8	364.5	60.8	320.5	259.3	179.7	159.3	97.3	312.9	229.2	205.0	150.1	143.4

	Condition W														
(Unit: mg/kg)	Manufacturer D					Manufacturer E					Manufacturer F				
Metabolite	Raw material	Intermediate	Final pickle	Initial rice-bran bed	Final rice-bran bed	Raw material	Intermediate	Final pickle	Initial rice-bran bed	Final rice-bran bed	Raw material	Intermediate	Final pickle	Initial rice-bran bed	Final rice-bran bed
Glutamic acid	70.1	114.4	4149.5	355.7	3734.5	69.1	175.3	7043.3	695.9	5650.5	171.1	63.9	560.8	319.6	445.4
Lactic acid	202.7	205.2	9889.3	1120.4	8804.2	184.5	407.6	702.7	226.2	1054.8	306.8	71.1	2423.1	7.2	249.8
